# GJA1-20k and Mitochondrial Dynamics

**DOI:** 10.3389/fphys.2022.867358

**Published:** 2022-03-23

**Authors:** Daisuke Shimura, Robin M. Shaw

**Affiliations:** Nora Eccles Harrison Cardiovascular Research and Training Institute, University of Utah, Salt Lake City, UT, United States

**Keywords:** GJA1-20k, connexin43, mitochondria, ischemia, trafficking, actin

## Abstract

Connexin 43 (Cx43) is the primary gap junction protein of mammalian heart ventricles and is encoded by the gene *Gja1* which has a single coding exon and therefore cannot be spliced. We previously identified that *Gja1* mRNA undergoes endogenous internal translation initiated at one of several internal AUG (M) start codons, generating N-terminal truncated protein isoforms that retain the C-terminus distal to the start site. GJA1-20k, whose translation initiates at mRNA M213, is usually the most abundant isoform in cells and greatly increases after ischemic and metabolic stress. GJA1-20k consists of a small segment of the last transmembrane domain and the complete C-terminus tail of Cx43, with a total size of about 20 kDa. The original role identified for GJA1-20k is as an essential subunit that facilitates the trafficking of full-length Cx43 hexameric hemichannels to cell-cell contacts, generating traditional gap junctions between adjacent cells facilitating, in cardiac muscle, efficient spread of electrical excitation. GJA1-20k deficient mice (generated by a M213L substitution in *Gja1*) suffer poor electrical coupling between cardiomycytes and arrhythmogenic sudden death two to 4 weeks after their birth. We recently identified that exogenous GJA1-20k expression also mimics the effect of ischemic preconditioning in mouse heart. Furthermore, GJA1-20k localizes to the mitochondrial outer membrane and induces a protective and DRP1 independent form of mitochondrial fission, preserving ATP production and generating less reactive oxygen species (ROS) under metabolic stress, providing powerful protection of myocardium to ischemic insult. In this manuscript, we focus on the detailed roles of GJA1-20k in mitochondria, and its interaction with the actin cytoskeleton.

## Introduction

**FIGURE 1:**
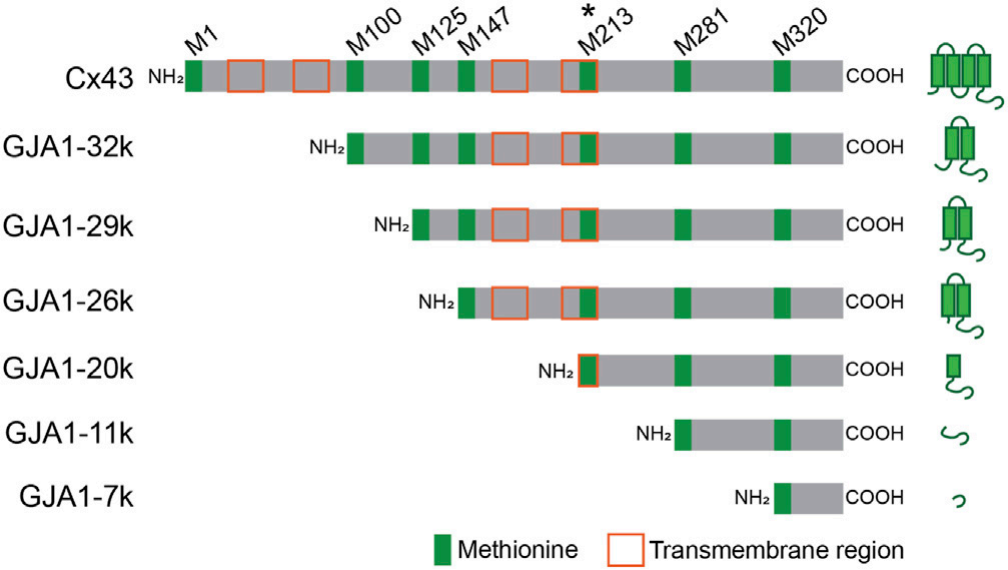
Schematic Gja1 mRNA of full-length Cx43 and N-terminus truncated isoforms. GJA1-20k is translated from start codon at M213 and most abundant isoform. Green and orange squares indicate methionine on Gja1 mRNA and transmembrane region after translation, respectively.

Gap junctions are specialized intercellular channels on outer cell membranes, providing low resistance communication between adjacent cells where they permit the rapid exchange of small molecules such as ions and metabolites. In the heart, especially in ventricular muscle, Connexin 43 (Cx43), which contains four transmembrane regions inclusive of residues 24–44, 77–97, 156–176, and 208–228; is the dominant gap junction protein forming channels that are composed of the serial coupling of two hexameric hemichannels (connexons), one across each adjoining cell membrane (Beyer et al., 1987; Yancey et al., 1989; Yeager and Gilula, 1992). Cx43 gap junctions in the heart have an essential role in maintaining proper intracardiac spread of cardiac electrical activity. *Gja1* is the gene encoding protein Cx43. Because *Gja1* contains a single coding exon, the gene cannot be subjected to splicing and there is no transcriptional variation in formation of Gja1 coding mRNA. Interestingly however, *Gja1* mRNA contains six internal AUGs (Methionine; M100, M125, M147, M213, M281, M320) which function as internal translation start codons. Up-to six N-terminal truncated small isoforms can be formed with occurrence of internal translation (Smyth and Shaw, 2013; Salat-Canela et al., 2014; Ul-Hussain et al., 2014). We have named the internally translated isoforms after their predicted size based on start codon location, specifically GJA1-32k, GJA1-29k, GJA1-26k, GJA1-20k, GJA1-11k, and GJA1-7k, respectively (Smyth and Shaw, 2013). GJA1-32k, GJA1-29k, GJA1-26k, and GJA1-20k have at least part of a transmembrane region and the complete C-terminus tail of Cx43 (Figure 1). GJA1-11k and GJA1-7k consist of terminal ends of the Cx43 C-terminus.

GJA1-20k is typically the most abundant endogenous internally translated isoform and is essential as a trafficking subunit for full-length Cx43 hemichannel movement to cell-cell borders (Smyth and Shaw, 2013; Xiao et al., 2020). These data were initially found in cell lines (Smyth and Shaw, 2013). Then, to further explore the role GJA1-20k, we generated a mouse line in which M213 is mutated to Leucine (M213L), which limits GJA1-20k translation yet retains full-length Cx43 translation (Xiao et al., 2020). The *in vivo* studies using our mouse line confirmed that a lack of GJA1-20k reduces formation of gap junctions. Furthermore, without GJA1-20k, poorly trafficked Cx43 does not reach the safety of its membrane domain, and is subject to increased Cx43 protein degradation (Xiao et al., 2020).

In a role distinct from hemichannel trafficking, we have also found that GJA1-20k localizes to mitochondrial outer membrane where it regulates mitochondrial morphology and metabolic function. Endogenous GJA1-20k increases with oxidative and ischemic stress (Fu et al., 2017; Basheer et al., 2018; Shimura et al., 2021a) and protects cells and organs against stress induced death. The mechanism of GJA1-20k induced ischemic protection is based on its effect on mitochondrial dynamics. Mitochondria undergo recurrent fission (fragmentation) and fusion (elongation), which is an adaptation to adjust to changing metabolic circumstances (Schrepfer and Scorrano, 2016). Canonical mitochondrial fission is associated with DRP1 and the degradation (breakdown) of dysfunctional and damaged mitochondria (Jheng et al., 2012; Friedman and Nunnari, 2014). In contrast, we have found that GJA1-20k induces a protective form of mitochondrial fission (Shimura et al., 2021a). Interestingly, GJA1-20k also stabilizes actin cytoskeleton. GJA1-20k stabilization of actin is likely the common denominator of GJA1-20k divergent roles from trafficking to mitochondrial regulation (Basheer et al., 2017; Shimura et al., 2021a).

## Full-Length CX43 Trafficking

### GJA1-20k Expression and Function *In vitro*

After Cx43 translation and post-translational modification in the endoplasmic reticulum (ER), Cx43 oligomerizes into hexamers as gap junction hemichannels in the Trans-Golgi Network (TGN) (Musil and Goodenough, 1993; Hussain et al., 2018). The hemichannels are then trafficked to cell membrane at cell-cell borders along microtubule “highways” (Shaw et al., 2007; Basheer and Shaw, 2016). Meanwhile, GJA1-20k is also internally translated beginning at the M213 start codon on *Gja1* mRNA. Because GJA1-20k does not contain a full transmembrane domain, it does not generate hemichannels itself but rather the C-terminus with a small portion of the last transmembrane domain. GJA1-20k functions as a necessary auxiliary trafficking subunit for full-length Cx43, organizing the actin cytoskeleton to pattern microtubule delivery highways (Smyth et al., 2012; Basheer et al., 2017).

It has been observedthat the C-terminal tail of Cx43, which includes GJA1-20k, has several phosphorylation sites and that Cx43 phosphorylation regulates Cx43 gap junction formation and stabilization (Laird, 2005; Moreno, 2005; Procida et al., 2009; Rodriguez-Sinovas et al., 2021). In addition, post-translational modification such as phosphorylation and dephosphorylation occurs during ischemia or reperfusion stress (Beardslee et al., 2000; Rusiecka et al., 2020). For instance, phosphorylation at serine 368 at C-terminal of Cx43 *via* Sphingosine-1-phosphate (Morel et al., 2016) occurs, or activation of the ERK pathway affecting Cx43 phosphorylation at serine 262/279/282 (Solan et al., 2019) both reduce damage from ischemia-reperfusion (I/R) injury. However, it remains to be explored whether post translational modification of GJA1-20k affects its function.

Endogenous GJA1-20k expression has been reported in not just cardiomyocytes, but also in various cell types such as C33A, NF-1, MEF, A549, HEK293 (Salat-Canela et al., 2014; Basheer et al., 2018; Shimura et al., 2021a). In all these cell types, GJA1-20k expression is the most abundant among the N-terminus truncated small isoforms of Cx43. The translation of GJA1-20k can be affected by inhibition of metabolic pathways including PI3K/AKT/mTOR and Mnk1/2 (Smyth and Shaw, 2013; Salat-Canela et al., 2014).

### Role of GJA1-20k Mediated Trafficking in a Gja1^M213L/M213L^ Mouse Model

In addition to the *in vitro* trafficking role identified for GJA1-20k, it is helpful to analyze its role *in vivo* using a mouse model. We recently generated a mouse line which has a Methionine (M) to Leucince (L) point mutation at codon M213 on gene *Gja1* (Gja1^M213L/M213L^ mouse), achieved by use of a CRISPR/Cas9 gene editing system (Xiao et al., 2020; Shimura et al., 2022). Due to the M213L mutation GJA1-20k is not translated from *Gja1* mRNA of the homozygous mouse, yet full-length Cx43 expression is preserved. Interestingly, mice with the homozygous mutation (Gja1^M213L/M213L^) always die suddenly within two to 4 weeks post-natal, whereas mice with the heterozygous mutation (Gja1^M213L/WT^ mouse) maintain near normal cardiac function and similar life span to that of wild type animals (WT) (Xiao et al., 2020). Therefore, the homozygous mouse line has a young-adult age-limitation for analysis yet the heterozygous animals can be studied well into adulthood. In young homozygous Gja1^M213L/M213L^ mouse hearts, gap junction formation is significantly reduced and full-length Cx43 remains in the cytosol, indicating a lack of Cx43 trafficking by GJA1-20k. Moreover, cytosol-retained Cx43 without GJA1-20k degrades faster than with GJA1-20k, indicating that GJA1-20k mediated trafficking to intercalated discs prevents Cx43 protein degradation.

In mammalian hearts, include human hearts, the expression level of GJA1-20k is enhanced by ischemic stress, indicating that GJA1-20k is a stress-responsive peptide (Basheer et al., 2018). In addition to loss-of-function analysis using Gja1^M213L/M213L^ mutant mouse line, gain-of-function of exogenous GJA1-20k introduced *in vivo* has been investigated. Adeno-associated virus type 9 (AAV9) is useful vector for specific gene delivery to cardiomyocytes (Inagaki et al., 2006). Introduction of AAV9 vectors by retro-orbital injection enables us to express genes, include GJA1-20k, into cardiomyocytes (Ahmed et al., 2013; Basheer et al., 2017). In contrast to GJA1-20k deficient (Gja1^M213L/M213L^) mouse hearts, AAV9-induced GJA1-20k overexpressed mouse hearts increase Cx43 gap junction plaques at the intercalated discs (Basheer et al., 2017). It has been reported that acute ischemic stress disrupts Cx43 gap junction plaque formation (Smyth et al., 2012). Interestingly, AAV9-induced GJA1-20k overexpression in heart maintains Cx43 gap junction plaque after acute ischemia injury, while full-length Cx43 overexpression by AAV9 is insufficient to rescue the plaque disruption by ischemia injury (Basheer et al., 2017).

Taken together, internally translated peptide “GJA1-20k” is essential for full-length Cx43 protein trafficking to cell membrane and maintains gap junction stability in stress situations such as cardiac ischemia. A lack of GJA1-20k causes gap junction formation deficiency in cardiomyocytes, Cx43 degradation in the cytosol, and arrhythmogenic sudden death.

## Actin Dynamics

### Actin Stabilization by GJA1-20k

Cx43 trafficking requires not only microtubule highways but also actin cytoskeleton as protein rest-stops and microtubule guides (Smyth et al., 2012; Basheer and Shaw, 2016). As a trafficking subunit, GJA1-20k interacts with both microtubules (see *Mitochondria Regulation by GJA1-20K*) and actin. In particular, GJA1-20k interacts with the actin cytoskeleton by stabilizing f-actin polymerization (Basheer et al., 2017). Although overall actin protein expression level is not altered by GJA1-20k, f-actin fiber length and number are increased by exogenous GJA1-20k overexpression in both HeLa cell and cardiomyocytes (Basheer et al., 2017). Actin polymerization by GJA1-20k supports microtubules orientation toward the cell-cell borders, permitting gap junction formation (Basheer and Shaw, 2016; Basheer et al., 2017). Interestingly, GJA1-20k does not promote actin polymerization directly. Instead, GJA1-20k inhibits actin depolymerization, resulting in a net effect of increased polymerization (Shimura et al., 2021a). Latrunculin A (LatA) disrupts actin fibers and limits Cx43 localization to the intercalated discs (Coue et al., 1987; Smyth et al., 2012). Polymerized actin regulated by GJA1-20k is resistant to LatA treatment supporting GJA1-20k′s role as a depolymerization inhibitor (Basheer et al., 2017; Shimura et al., 2021a).

### How Does GJA1-20k Interact With Actin?

It has been previously reported that actin can be co-immunoprecipitated with GJA1-20k, suggesting that GJA1-20k and actin complex (Basheer et al., 2017). However, it remains unclear whether GJA1-20k directly binds to actin or not. The RPEL domain, which is defined as RPxxxEL, is a well-known as an actin binding motif (Miralles et al., 2003). Our recent data find that a RPEL-like actin binding motif, RPRPDDLEI, at the C-terminus of GJA1-20k is an actin binding site (preprint available on BioRxiv) (Baum et al., 2022). In this study, we identified that GJA1-20k transfected cells produce not only thickened actin filaments but also actin puncta. Importantly, these puncta disappear with *Gja1* knock-down and are rescued by introduction of exogenous GJA1-20k but not full-length Cx43 (Baum et al., 2022). These data indicate that GJA1-20k itself, but not full-length Cx43, has responsibility for actin filament formation and stability. The existence of puncta is a phenotype of similar to actin capping-proteins (CPs) which are well-known regulators of actin polymerization and depolymerization (Schafer et al., 1995; Cooper and Sept, 2008). It is an intriguing possibility than GJA1-20k interacts directly with actin and functions similar to CPs (Baum et al., 2022).

Given that GJA1-20k has an identical sequence to the tail of Cx43, it is not clear why GJA1-20k induces profoundly different cellular phenotypes and functions. We expect that a key reason is that GJA1-20k can exist in an aqueous domain and even potentially dimerizes (Baum et al., 2022). Therefore, GJA1-20k is not restricted to a larger hexameric channel and, when in the cytoplasm, is not restricted to a vesicle carrying multiple copies of the larger connexon ion channel. In a sense, GJA1-20k is the more available (and more potent) form of the Cx43 tail because it is a peptide that is literally unleashed from the restrictions of being connected to large hexameric lipid bound ion channel.

## Mitochondria Regulation by GJA1-20K

### Mitochondrial Co-Localization and Transport by GJA1-20k

It has been reported that full-length Cx43 is localized not only at surrounding cell membrane for formation of intercellular gap junctions but also at the mitochondrial inner membrane, mediated by heat shock protein 90 (HSP90) and TOM pathway (Rodriguez-Sinovas et al., 2006; Rodriguez-Sinovas et al., 2018). At mitochondrial inner membrane, Cx43 could form hemichannels and regulate mitochondrial respiration by modulating potassium ion flux and respiratory complex activity (Miro-Casas et al., 2009; Boengler et al., 2012; Rodriguez-Sinovas et al., 2018). Interestingly, mitochondria in cardiomyocytes can be categorized into two groups depending on their cellular localization and subpopulation; the subsarcolemmal (SSM) and the interfibrillar (IFM) mitochondria. SSM mitochondria are more responsible to stress compared to IFM mitchondria (Palmer et al., 1977; Kavazis et al., 2009). The mitochondrial full-length Cx43 in cardiomyocyte is mostly restricted to be in SSM mitochondria (Boengler et al., 2009), suggesting stress-related functions of full length Cx43 in mitochondria.

**FIGURE 2:**
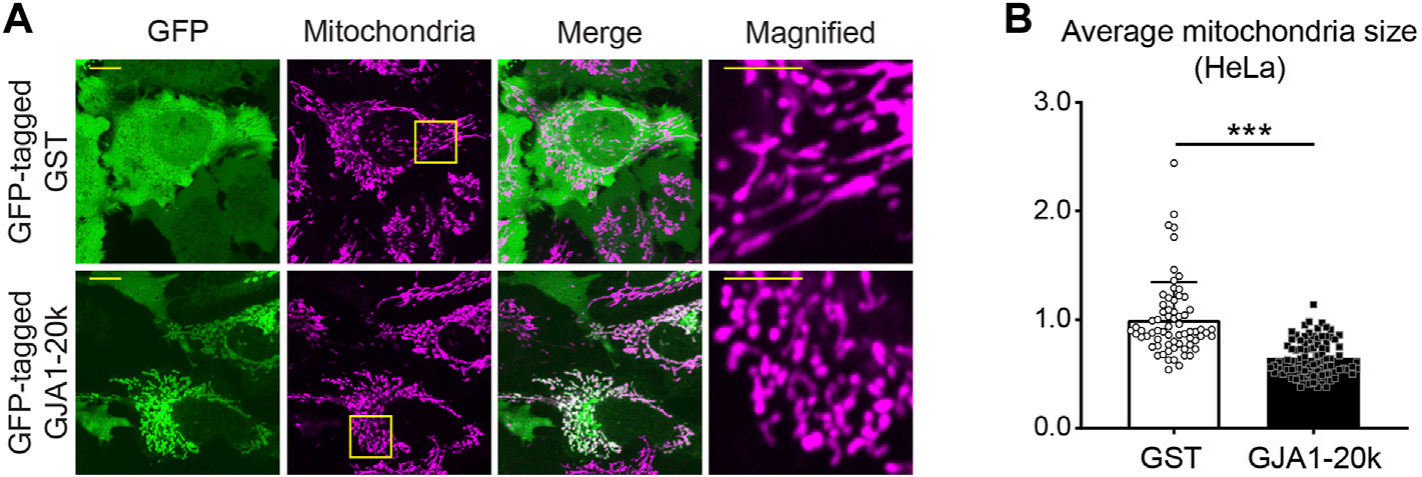
GJA1-20k locates at mitochondria and induces mitochondrial fission. **(A)** The representative live-cell images of GFP-tagged GJA1-20k (or GST as control) transfected HeLa cells. Mitochondria are visualized by mitotracker. The panels on the right indicate magnified images. Scale bars, 10 μm and 5 μm in magnified images. **(B)** The quantified fold change in the average mitochondria size with or without exogenous GJA1-20k. *n* = 68 (GST) or 89 (GJA1-20k) cells. Graphs are expressed as mean ± SD. *p* value is determined by two-tailed Mann-Whitney *U*-test. ***p < 0.001.

As well as full-length Cx43, GJA1-20k is also localized mitochondria, whereas the localization is on the outer membrane of mitochondria (Fu et al., 2017; Basheer et al., 2018). Although there is little knowledge of the GJA1-20k localization to different mitochondrial subpopulations, an early report indicated GJA1-20k localizes to SSM mitochondria in cardiomyocytes (Basheer et al., 2018). In addition, the co-localization between GJA1-20k and mitochondria can be seen not only in cardiomyocytes (Basheer et al., 2018) but also in several cell lines such as HaCaT cells, HEK293 cells, Glial cells, Cardiac fibroblasts, and HeLa cells (Fu et al., 2017) (Figure 2A).

The originally identified role of GJA1-20k in mitochondria was to promote microtubule-dependent mitochondrial transport (Fu et al., 2017). Importantly, GJA1-20k has a microtubule-binding domain (MTBD; 234th to 259th amino acids of Cx43) (Saidi Brikci-Nigassa et al., 2012) and conveys mitochondria along with microtubules similar to the transport of Cx43 hemichannels (Fu et al., 2017). Overexpression of GJA1-20k increases the velocity and the net displacement of mitochondria movement in cells (Fu et al., 2017). It is also known that full-length Cx43 also binds to microtubules and regulate its trafficking to cell membrane on microtubule “highway” (Epifantseva and Shaw, 2018), however, MTBD of full-length Cx43 has not been reported to be involved in mitochondrial trafficking.

Mitochondria are overall well distributed in the cells but constantly and dynamically travel throughout the cytosol *via* the microtubule network and use of motor proteins. The net distribution can be altered by cellular circumstances (Frederick and Shaw, 2007; Saotome et al., 2008). For instance, H_2_O_2_ treatment can add oxidative stress to cells and mimic ischemic stress. H_2_O_2_ stress induces mitochondrial shrinkage and centralization but exogenous GJA1-20k transfection rescues the mitochondrial distribution, suggesting one method by which GJA1-20k help resist against ischemic stress (Fu et al., 2017). Furthermore, it has been recently reported that GJA1-20k in astrocytes transport mitochondria to neurons through Cx43 hemichannels (Ren et al., 2021). This mitochondria delivery to neurons results in the protective repair of damaged neurons under traumatic brain injury. The astrocyte-delivered mitochondria also promote mitochondrial biogenesis, suggesting an additional role for GJA1-20k as a protective mitochondrial regulator (Ren et al., 2021).

### GJA1-20k Induces Protective Mitochondrial Fission

Mitochondria are dynamic organelles which repeat fission (fragmentation) and fusion (fusion). Not just its distribution, but individual mitochondrial morphology can reflect cellular circumstance (Youle and van der Bliek, 2012; Friedman and Nunnari, 2014). However, it is no longer canonical thinking the fission is bad for the cell and fusion is good for the cell. Mitochondrial morphology does not reflect cell status, but rather it is important to consider how mitochondrial morphology was determined.

Since GJA1-20k is localized at mitochondrial outer membrane, we have recently focused on the effects of GJA1-20k on mitochondrial morphology and identified that GJA1-20k induces mitochondrial fission (Figure 2) and that GJA1-20k-induced smaller mitochondria suppress the generation of reactive oxygen species (ROS) under oxidative stress (Shimura et al., 2021a). From both *in vitro* and *in vivo* studies, exogenously overexpressed GJA1-20k induces smaller mitochondria and increases the number of mitochondria. In contrast, GJA1-20k deficient mouse cardiomyocytes showed elongated mitochondria (Shimura et al., 2021a). Generally, mitochondrial fission dynamics are regulated by well-known GTPase mediators. In mammalian cells, Dynamin-Related Protein 1 (DRP1) and Dynamin 2 (DNM2), are a main mediators of mitochondrial fission (Friedman and Nunnari, 2014; Lee et al., 2016; Kamerkar et al., 2018). In particular, DRP1 activity is regulated by its phosphorylation (Sabouny and Shutt, 2020).

Interestingly, GJA1-20k does not altered the expression and phosphorylation of DRP1 (Shimura et al., 2021a). Typically, deficiency of DRP1 or DNM2, and the presence of DRP1 dominant negative (K38A) induce mitochondria elongation due to fission inhibition. To our surprise, GJA1-20k-induced mitochondrial fission can be observed even under DRP1 or DNM2 knock-down and K38A transfection, indicating that mitochondrial fission mediated by GJA1-20k is independent or at least downstream of a canonical DRP1-mediated fission pathway (Shimura et al., 2021a). In addition, DRP1-mediated mitochondrial fission is sometimes associated with membrane permeability modification, cytochrome c release, and apoptosis (Oettinghaus et al., 2016; Milani et al., 2019). However, importantly, GJA1-20k does not affect mitophagy or membrane potential, suggesting that GJA1-20k induces mitochondrial fission but not degradation (Shimura et al., 2021a).

### Actin Involvement in GJA1-20k Mediated Mitochondrial Dynamics

As described in *GJA1-20k Induces Protective Mitochondrial Fission* above, the canonical understanding is that the main mediator of mitochondrial fission is DRP1, whereas GJA1-20k does not require the canonical DRP1 pathway to induce mitochondrial fission. Then, how does GJA1-20k induce mitochondrial fission without DRP1? In addition to DRP1, actin is necessary to finalize mitochondrial fission (De Vos et al., 2005; Li et al., 2018). It has been reported that actin-associated mitochondria shifts the fission-fusion balance to more fission, while actin disassociation results in mitochondrial fusion (Moore et al., 2016). Moreover, there are several studies reporting DRP1-independent fission in cells undergoing protective mitophagy for mitochondrial quality control and adaptation to higher energy demand in which the actin is still involved even under absence of DRP1 (Stavru et al., 2013; Yamashita et al., 2016; Coronado et al., 2018). As mentioned in *Actin Dynamics*, GJA1-20k binds to actin to regulate cellular activity. As expected, actin expression is detectable in the mitochondrial fraction from GJA1-20k transfected cells and actin is clearly assembled as a shield surrounding mitochondria with GJA1-20k (Shimura et al., 2021a). Recently, actin dynamics have been captured, indicating that actin is assembled around GJA1-20k-associated mitochondria and consequently induces fission (Shimura et al., 2021a). The relationship between actin assembly and mitochondrial fission in GJA1-20k transfected cells is consistent with previously reported actin association with mitochondria fission (Moore et al., 2016).

To the best of our knowledge, DRP1-independent mitochondrial fission has been reported but not actin-independent fission. For instance, actin disruption causes mitochondrial fusion and elongation (Li et al., 2018). As mentioned in *Actin Dynamics*, GJA1-20k stabilizes actin filaments and prevent actin disruption by LatA (Basheer et al., 2017; Shimura et al., 2021a). Therefore, GJA1-20k could inhibit mitochondrial elongation under LatA treatment by stabilizing the actin network around mitochondria (Shimura et al., 2021a).

**FIGURE 3:**
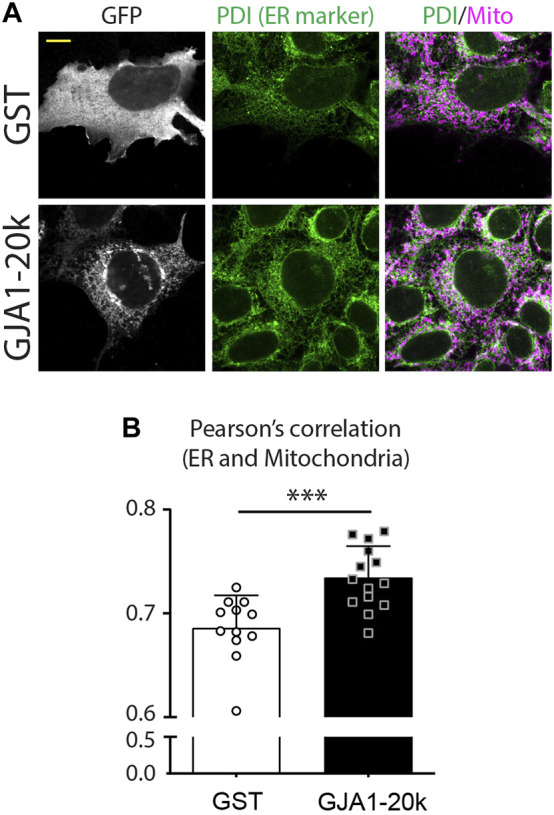
GJA1-20k increases interaction between ER and mitochondria. **(A)** Representative fixed-cell images of ER and mitochondria in GFP-tagged GJA1-20k (or GST as control) transfected HeLa cells. ER and Mitochondria are visualized by PDI and Tom20 antibodies, respectively. Scale bars, 10 μm. **(B)** The quantified Pearson’s correlation between ER and Mitochondria with or without exogenous GJA1-20k. *n* = 12 (GST) or 14 (GJA1-20k) cells. Graphs are expressed as mean ± SD. *p* value is determined by two-tailed Mann-Whitney *U*-test. ***p < 0.001. **These figures are published in Reference (Shimura et al., 2021b) as a response to Reviewer’s comments for the corresponding eLife publication (Shimura et al., 2021a).

As with actin dynamics for finalizing mitochondrial fission, there are also studies indicating that the endoplasmic reticulum (ER) is essential for fission (Li et al., 2018). ER also interacts with actin and DRP1 at mitochondrial fission points to induces the final stages of fission. Although it is unknown whether GJA1-20k directly interacts with ER or whether ER is involved in GJA1-20k induced mitochondrial fission, we recently find an increased ER network in GJA1-20k overexpressed cells (Figure 3). These data suggest the ER involvement into GJA1-20k could help induce mitochondrial fission, but further mechanistic investigation is required.

### Protective Metabolic Effects of GJA1-20k

As much as 40–50% of heart disease is related to ischemia (Wong, 2014). Ischemic heart disease is associated with a high incidence of mortality and morbidity due to acute arrhythmic sudden cardiac death and also to development of chronic heart failure. For acute ischemia from epicardial artery occlusion, revascularization is the treatment of choice. However, paradoxically, revascularization exacerbates damage to ischemic tissue by inducing excessive oxidative stress, which is referred to as ischemia-reperfusion (I/R) injury.

The phenomenon of ischemic preconditioning (IPC) was first described more than 30 years ago in the canine hearts (Murry et al., 1986). IPC occurs when is induced resistance to ischemic stress by antecedent shorter bouts of ischemia prior to a longer period of ischemia. IPC has been confirmed in not only the heart but also in organs such as the brain, liver, and kidney. In spite of many efforts for the therapeutic application of IPC, little the central mechanism that underlies IPC has been elusive, and clinical trials to date have not succeeded in reproducing IPC effects (Heusch and Gersh, 2020). During IPC, the phosphorylation of Serine 368 at C-terminal Cx43 and the HSP90/TOM pathway for Cx43 mitochondrial transport are activated which increase mitochondrial Cx43 (Boengler et al., 2005; Rodriguez-Sinovas et al., 2006; Miura et al., 2010; Rusiecka et al., 2020). Although the function of mitochondrial Cx43 is not fully understood, it has been reported that increases in Cx43 *via* phosphatidylinositol-3-kinase (PI3K)/Akt pathway (e.g., by Atorvastatin administration) protects the heart against I/R stress (Bian et al., 2015). Increases in mitochondrial Cx43 also enhances mitochondrial ATP-sensitive potassium (KATP) channels and is involved in potassium flux restricts ROS production during stress (Boengler et al., 2013; Soetkamp et al., 2014; Bian et al., 2015) providing preconditioning protection. Since Cx43 deficient hearts lose their protective effects against ischemic stress, full length mitochondrial Cx43 could participate in cardioprotection (Heinzel et al., 2005).

The Delmar research group has highlighted the protective effect role of C-terminus of Cx43 against ischemic stress (Maass et al., 2009). GJA1-20k contains full C-terminus tail of Cx43 (from the 213 residue) and, interestingly, post-ischemic mouse and human heart tissue showed increased in the expression level of GJA1-20k, indicating that GJA1-20k is a stress-responsive peptide (Basheer et al., 2018). As similar to the protective effect of Cx43-CT, AAV9-induced GJA1-20k overexpression in mouse hearts reduces infarct size by I/R injury without alteration of baseline cardiac function (Basheer et al., 2018). This protective effect of GJA1-20k is consistent with the effect of IPC, suggesting that GJA1-20k mimics IPC-like protective effect. In contrast, GJA1-20k deficient (M213L mutation) mouse heart has severe damage from I/R injury (Shimura et al., 2021a). It appears highly likely that a critical mediator of IPC protection is the upregulation of GJA1-20k, protecting organs from subsequent longer ischemic periods. As mentioned above, this protective effect has a strong relation to mitochondrial function, since GJA-20k associated mitochondria suppress ROS production and maintain efficient metabolism (Shimura et al., 2021a). Post-ischemic estrogen administration increases the levels of Cx43 and GJA-20k in mitochondria, results in reducing the infarct size of the heart and rescuing mitochondrial membrane potential after I/R injury stress (Wang et al., 2019).

I/R injury can be observed not only in the heart but also in other organs such as brain, liver, kidney, and skeletal muscle. Not surprisingly full length Cx43 has a focus as a therapeutic target to limit I/R injury, especially in the heart and the brain (Schulz et al., 2015; Hou et al., 2016). Cx43 is also a primary gap junction component to connect astrocytes and neurons in the brain and considered to have protective effects against ischemic stroke (Liang et al., 2020). Interestingly, it was recently identified that GJA1-20k can contributes to the repair the injured neurons by facilitating mitochondrial transfer (Ren et al., 2020; Ren et al., 2021). Given the protective effects of GJA1-20k in the setting of cardiac I/R injury, we expect additional studies indicating that GJA1-20k will protect against I/R injury in the brain. In addition to I/R injury, it has been reported that GJA1-20k overexpression rescues gap junction formation, promotes mitochondrial metabolism, and suppresses ROS production in Angiotensin II-induced hypertrophic cardiomyocytes (Fu et al., 2021). Collectively, these studies indicate the possibility of GJA1-20k as protective general mitochondrial regulator in additional to what is already known about Cx43. Particulars such as the difference between Cx43 (inner membrane) and GJA1-20k (outer membrane) in mitochondria need to be explored further.

## Conclusion and Future Prospects

**FIGURE 4:**
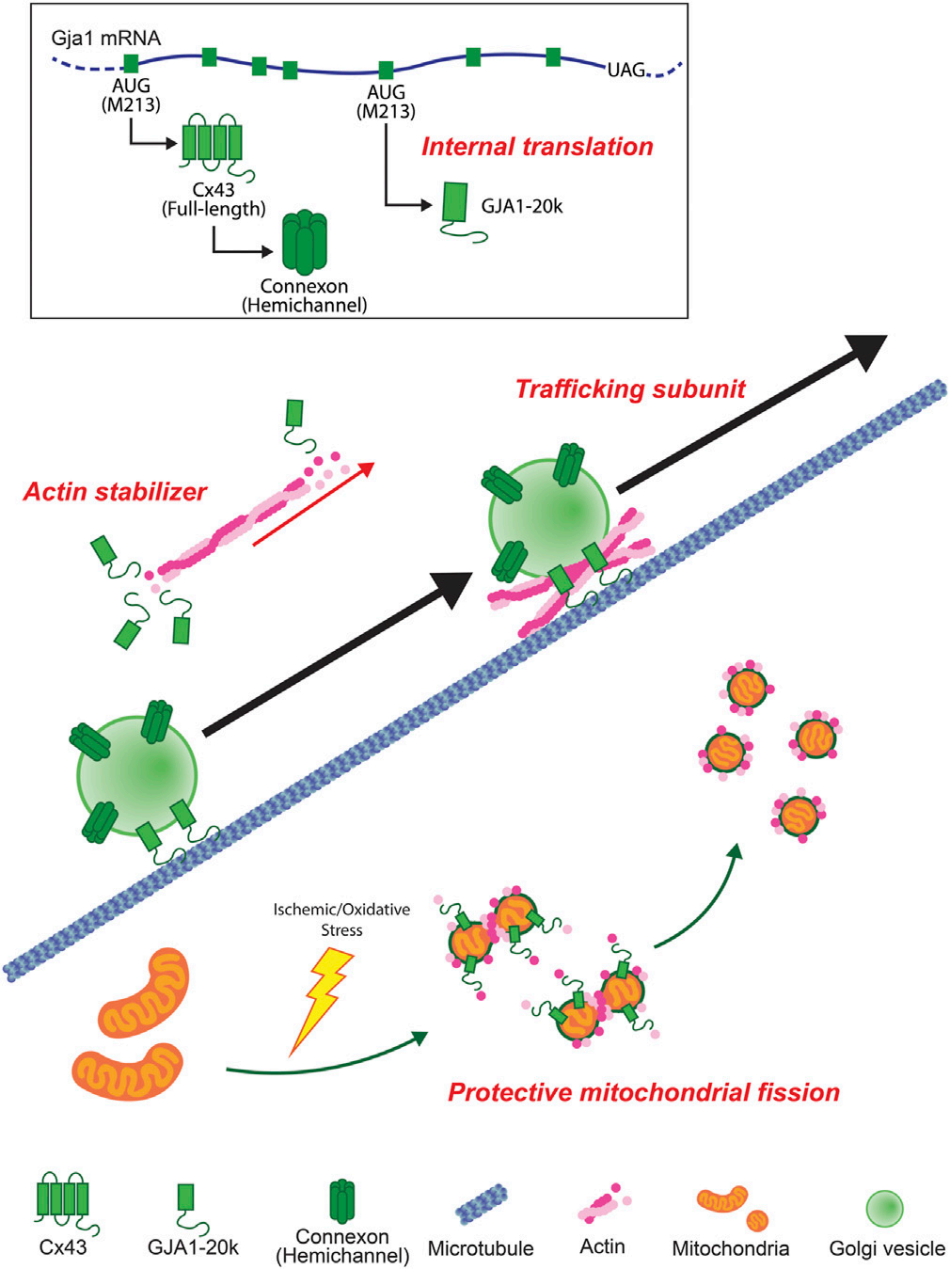
Schematic summary of GJA1-20k. GJA1-20k is generated by internal translation of Gja1 mRNA. GJA1-20k works not only as an auxiliary trafficking subunit for full-length Cx43 hemichannels to cell-cell borders but also as an actin stabilizer. In mitochondria, GJA1-20k is upregulated by ischemic/oxidative stress and works with actin to induce a protective mitochondrial fission.

In this review, we summarize some overall features of GJA1-20k which was only recently reported to exist endogenously (Smyth and Shaw, 2013). GJA1-20k has roles in cells that are distinct, and sometimes attributed to, full-length Cx43 such as regulation of the actin cytoskeleton, forward Cx43 trafficking, protective mitochondrial fission, and mediating ischemic preconditioning by protecting mitochondria against oxidative stress (Figure 4). While most studies involving GJA1-20k presently focus on the heart, GJA1-20k is present in other organs. As a gene therapy or, in the future, as a possible peptide, GJA1-20k has therapeutic potential against anticipated ischemia in the heart, brain, kidneys or other organs subjected to ischemic injury.
